# Supplementary material for: The Landscapes of Gluten Regulatory Network in Elite Wheat Cultivars Contrasting in Gluten Strength
Source: Int J Mol Sci. 2023 May 29;24(11):9447. doi: 10.3390/ijms24119447 (PMC10253585; doi:10.3390/ijms24119447)
Supplement: Supplementary file 1 [file ijms-24-09447-s001.zip › Figures S1-S4.pdf]

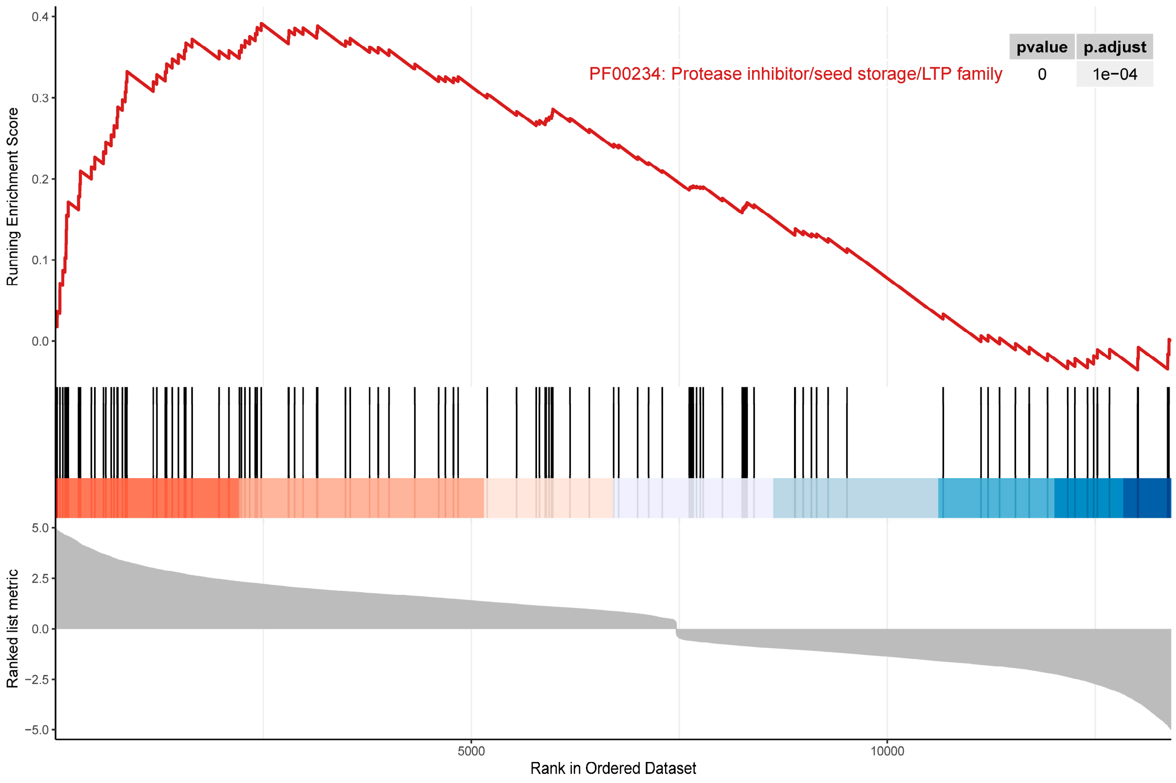

Figure S1: GSEA enrichment plot for protease inhibitor/seed storage/LTP family (PF00234).

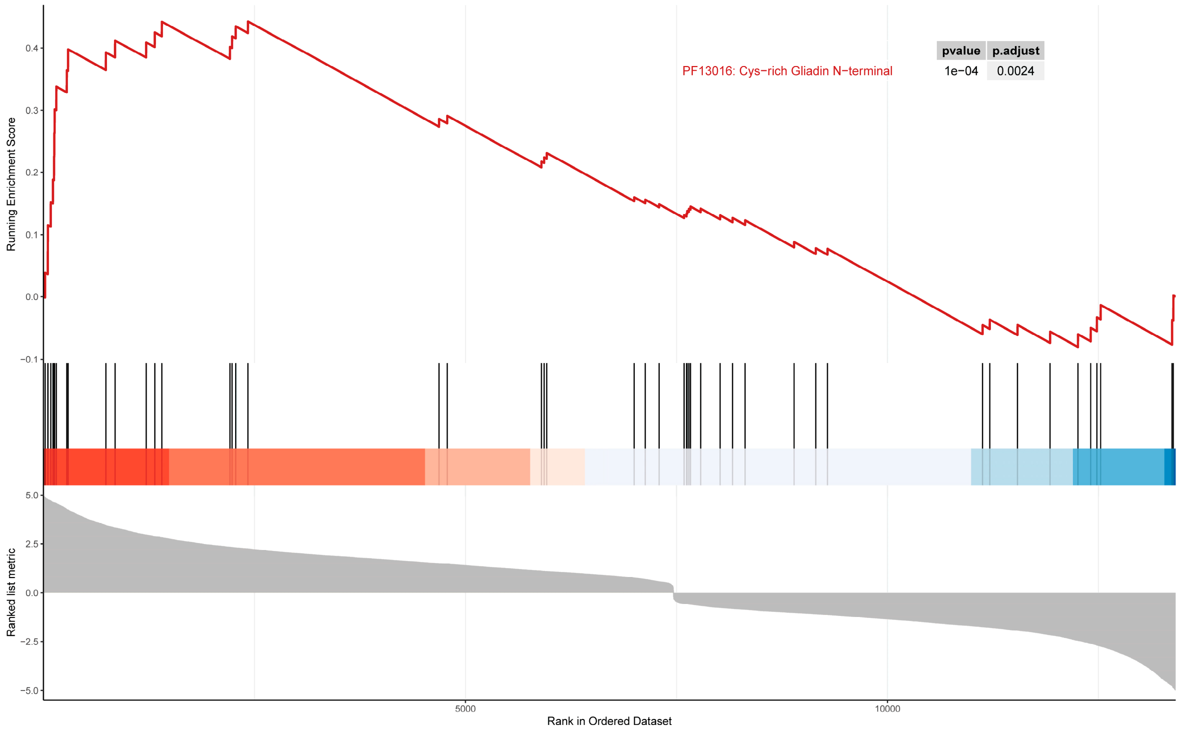

Figure S2: GSEA enrichment plot for Cys-rich Gliadin N-terminal (PF13016).

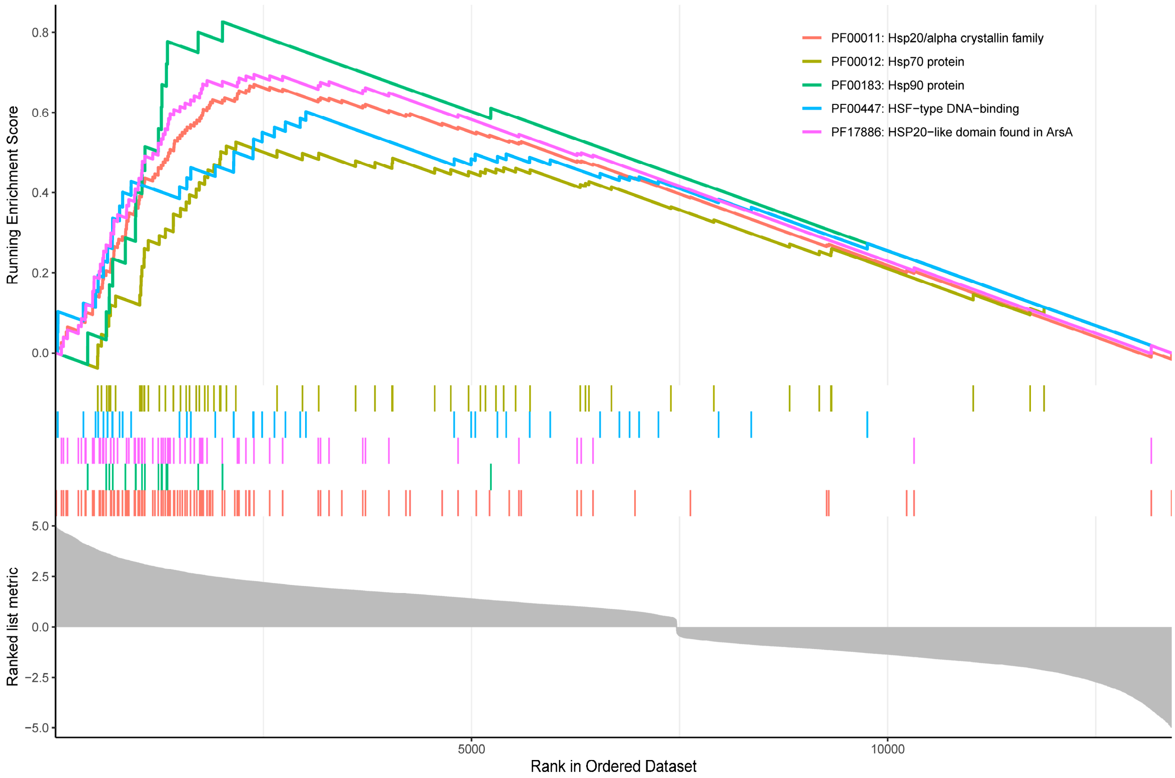

Figure S3: GSEA enrichment plot for HSP-related protein families.

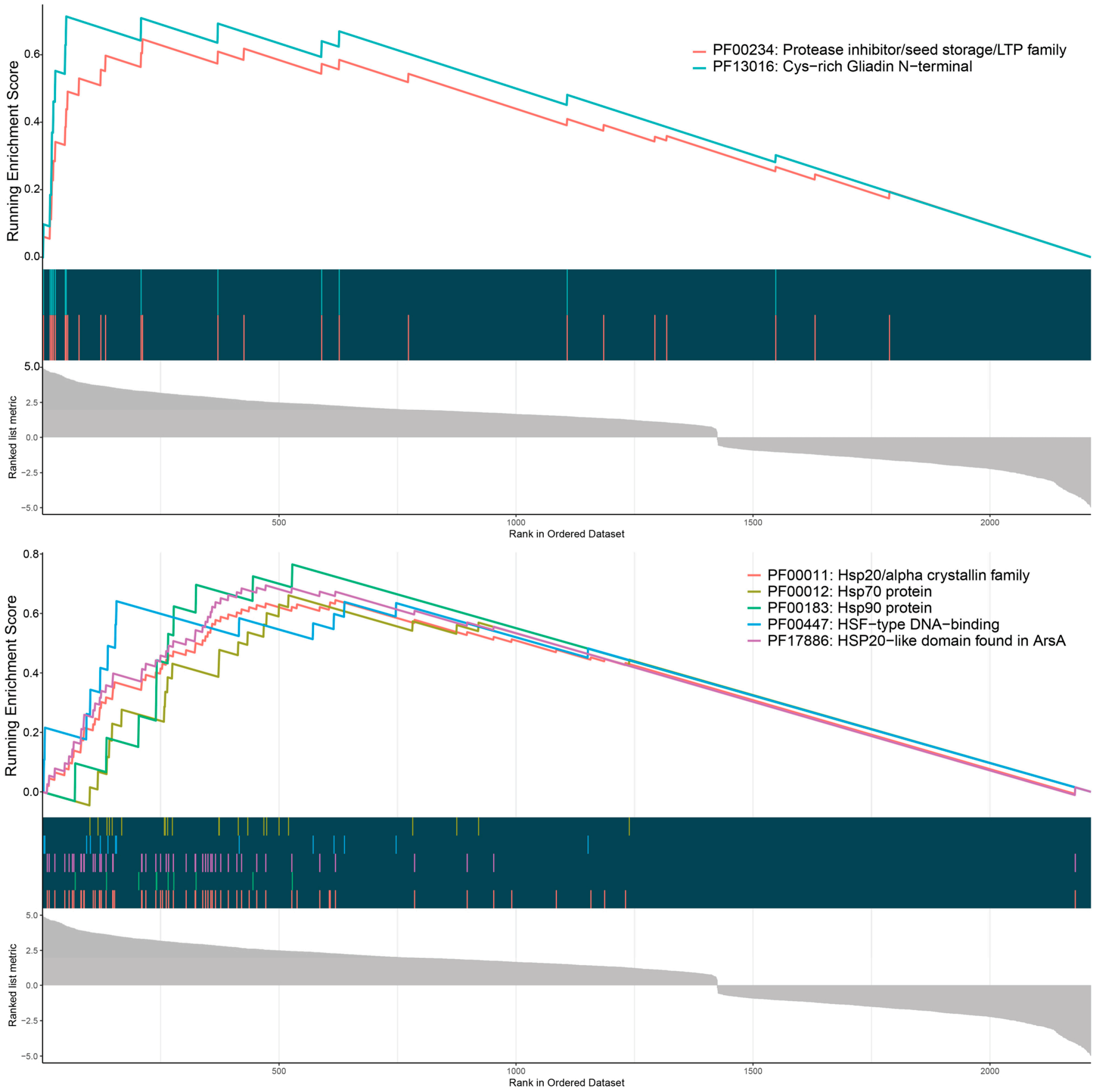

Figure S4: Highly enriched Pfam for genes in significant MEGENA modules
